# Primary Intraocular Lymphoma in a Patient with Bilateral Epstein-Barr Virus Panuveitis

**DOI:** 10.1155/2021/9496173

**Published:** 2021-10-06

**Authors:** Seyedeh Maryam Hosseini, Mojtaba Abrishami, Elham Barashki, Ghodsieh Zamani

**Affiliations:** Eye Research Center, Mashhad University of Medical Sciences, Mashhad, Iran

## Abstract

**Purpose:**

Herein, we report a case of primary intraocular lymphoma (PIOL) with the first presentation of bilateral Epstein-Barr virus- (EBV-) associated panuveitis. *Case Presentation*. A 69-year-old male was referred with a three-day history of blurred vision and pain and redness in his left eye following cataract surgery. Examination revealed panuveitis, vitritis, and necrotizing retinitis with retinal hemorrhage. A month later, the right eye was also involved. Polymerase chain reaction-based analysis of the vitreous sample was positive for EBV, and cytological evaluation was compatible with the diagnosis of B-cell lymphoma. A significant improvement was observed following serial intravitreal rituximab and methotrexate injections. The central nervous system and lungs were involved after 6 months, and the patient expired despite systemic chemotherapy.

**Conclusion:**

There may be an association between EBV panuveitis and primary intraocular B-cell lymphoma.

## 1. Introduction

Intraocular lymphoma is a rare form of ocular malignancy and includes two types, namely, primary and secondary. Primary intraocular lymphoma (PIOL) affects the vitreous, retina, choroid, or optic nerve without systemic lymphoma [1]. This disease has a poor prognosis with a 5-year survival rate of 61% [2]. The disease is typical of masquerade syndromes and frequent masquerades as chronic uveitis, and it is often difficult to diagnose if no characteristic vitreous haze or subretinal focus is present [3].

The PIOL is a rare and potentially fatal intraocular malignancy. More than half of the cases eventually involve the central nervous system (CNS). Optical coherence tomography (OCT) and fundus autofluorescence (FAF) have been applied for the diagnosis of PIOL. In addition, histology and immunohistochemistry, in combination with molecular tests and interleukin-10 analysis, have been demonstrated as reliable in its diagnosis [4]. The PIOLs are usually diffuse large B-cell lymphomas (DLBCL) with very few cases of primary T-cell lymphoma described in the literature [5]. Herein, we report a patient with PIOL primarily presenting with bilateral Epstein-Barr virus- (EBV-) associated panuveitis, accompanied by DLBCL, who was deceased due to CNS involvement 6 months following that time.

## 2. Case Presentation

A 69-year-old male was referred with left eye pain, redness, and poor vision 3 days after uncomplicated phacoemulsification and posterior chamber intraocular lens implantation. On presentation, the best-corrected visual acuity (BCVA) values were 20/32 and 20/800 in the right and left eyes, respectively. There was no relative afferent pupillary defect. The findings of the slit-lamp examination of the left eye were microcystic corneal edema, localized Descemet's detachment, and 3+ cell and fibrin in the anterior chamber.

**Figure 1:**
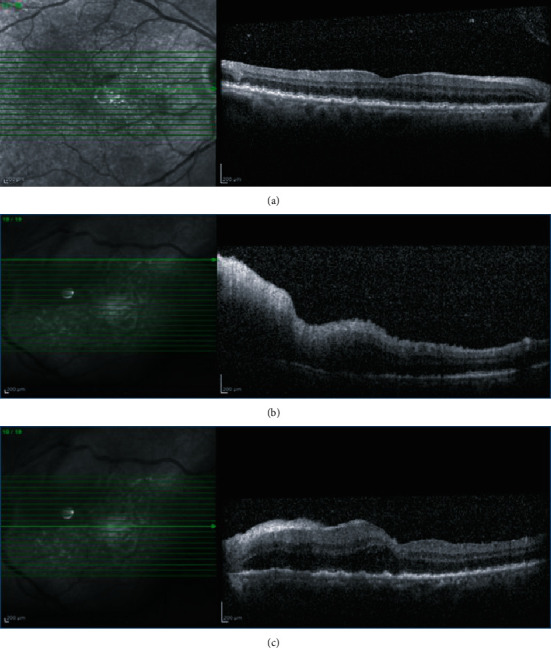
Spectral domain OCT (SD-OCT) at the first presentation. (a) Right eye: multiple tiny Drusen-like RPE elevations. (b, c) Left eye: retinal edema, increased central macular thickness (CMT), multiple RPE irregularity simulating Drusen, and inner retinal hyperreflective lesion indicating retinitis at the superotemporal of the optic disc.

Posterior segment examination disclosed vitritis, optic disk swelling, vascular sheathing, small intraretinal hemorrhages and patches of retinitis in the superior quadrant, and pigmentary changes at the macula in the left eye. The right eye appeared normal except for senile cataract and small Drusen-like lesions (Figure 1). Firstly, the clinical diagnosis of acute retinal necrosis was made. Therefore, undiluted vitreous sampling was prepared for polymerase chain reaction (PCR) evaluation of infectious etiologies, including herpes simplex virus (HSV), cytomegalovirus (CMV), varicella-zoster virus (VZV), EBV, and Toxoplasma. Moreover, vitreous and aqueous humor samples for smear and culture for bacterial infection with a probable diagnosis of acute postoperative endophthalmitis were also acquired.

The patient was treated with intravenous acyclovir (10 mg/kg/every 8 h), intravitreal ganciclovir (twice a week), and systemic oral prednisolone (50 mg/day). The retinitis and inflammation were partially improved following the treatment. After 10 days of treatment, systemic intravenous acyclovir was replaced by valacyclovir (1 g/every 8 h) with an incomplete response to the treatment.

**Figure 2:**
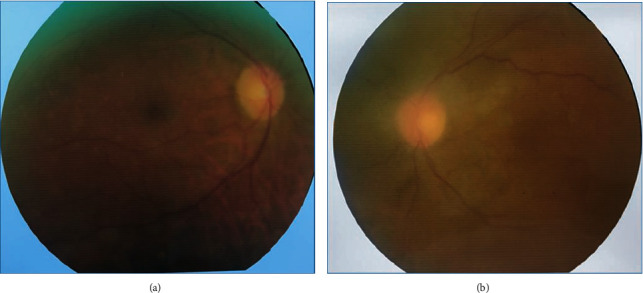
Color fundus photograph, one month after starting the antiviral therapy. (a) Right eye: multiple subretinal punctate lesions resembling Drusen scattered on the temporal side of the fovea. (b) Left eye: vitreous haziness, disc paleness, and resolving retinitis associated with pigmentary change at the macular area and superior to the disc.

**Figure 3:**
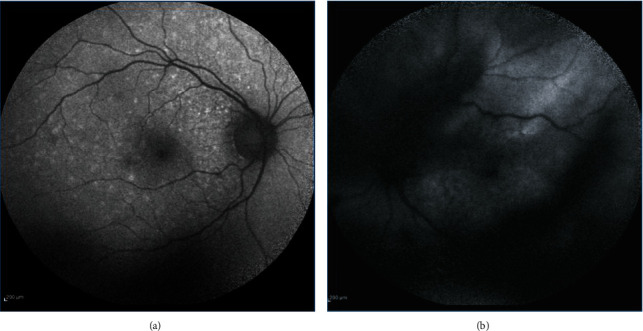
Fundus autofluorescence (FAF), one month after starting the antiviral therapy. (a) Right eye: numerous hyperautofluorescent dots scattered on the posterior pole without vitreous opacity. (b) Left eye: stippled mixed hyper-hypoautofluorescent dots (leopard pattern) at the posterior pole and hypoautofluorescent vitreous opacity corresponding to the vitritis.

A month later, the patient complained of decreasing vision. Vitreous reaction accompanied by retinal vasculitis and punctate Drusen-like subretinal infiltration foci were observed in the examination of the right eye (Figure 2). The FAF showed numerous hyperautofluorescent dots scattered at the posterior pole of the right eye and stippled mixed hyper- and hypoautofluorescent dots (leopard pattern) at the posterior pole and vitreous opacity corresponding to the vitritis in the left eye (Figure 3). Fluorescein angiography was not provided due to an allergic reaction. The aforementioned treatment was continued, and vitreous sampling of the right eye was performed for PCR and cytological evaluation with the suspicion of masquerade syndrome, especially PIOL.

**Figure 4:**
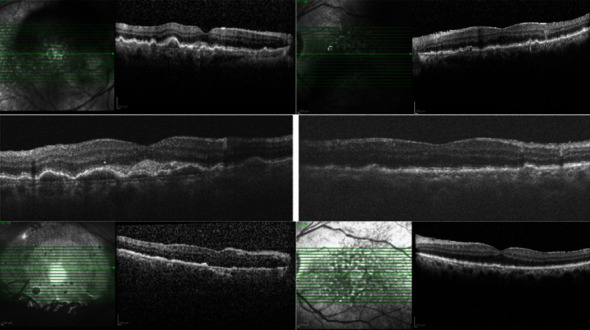
One month after starting the antiviral therapy, SD-OCT of the right eye (a) and the left eye (b) shows the exacerbation of the subretinal infiltration during the one-week follow-up period (the top and the middle rows). Two weeks after intravitreal injection of methotrexate and rituximab, significant improvement was observed in macular OCT (bottom row).

Spectral-domain optical coherence tomography (SD-OCT) revealed multiple tiny Drusen-like retinal pigmented epithelium elevation in the right eye and retinal edema, hyperreflectivity of inner retina at retinitis area, and increased central macular thickness in the left eye (Figure 4).

In the analysis of the vitreous sample, the results of PCR for VZV, HSV, CMV, and Toxoplasma were all negative, except for EBV deoxyribonucleic acid (DNA) in both eyes. In addition, the histopathological examination of the vitreous sample showed malignant lymphocyte cells compatible with the diagnosis of B-cell lymphoma with positive CD20 and CD19 markers. Systemic workups, including brain magnetic resonance imaging (MRI) with contrast enhancement and ultrasound of the pelvic, abdomen, and cervical lymph node chain, and oncologist consultation revealed no pathologic findings. As a result, with the diagnosis of PIOL, local intraocular chemotherapy (monthly intravitreal rituximab and methotrexate twice a week for 1 month, weekly for next month, and then monthly) was initiated. Clinical response was good (Figure 4), and BCVA increased to 20/32 and 20/50 in the right and left eyes, respectively.

After 4 months, symptoms of cognitive and behavioral impairment appeared. However, the second brain MRI was also normal; the protein level of cerebrospinal fluid (CSF) analysis was high. The cytological evaluation of the CSF sample revealed malignant lymphocyte cells. Therefore, with the diagnosis of CNS lymphoma, systemic chemotherapy was also initiated. The patient expired during systemic chemotherapy.

## 3. Discussion

Herein, we reported a case of PIOL and DLBCL associated with EBV intraocular infection in both eyes. The etiology of PIOL is not very clear. Two theories have been implicated in the etiology of PIOL, namely, (1) infectious theory and (2) hematological spread. Infections with EBV or human immunodeficiency virus (HIV), especially in immunocompromised patients, recall the lymphoid cells; however, neoplastic transformation to lymphoid malignancy occurs later at the eye and/or CNS [6]. The occurrence of EBV-induced lymphoid or epithelial malignancies is well recognized [7]. The EBV-associated DLBCL of the elderly is a subtype of DLBCL according to the 2008 classification of the World Health Organization. This is an extremely rare tumor, and to the best of our knowledge, few cases of ocular EBV-associated DLBCL in the elderly have been reported [8].

Mashima et al. [9] described an 83-year-old woman developing unilateral necrotizing retinitis. The PCR-based testing revealed high EBV DNA levels in the vitreous, but not in the plasma. Testing of the vitreous for HSV, VZV, and CMV DNA was all negative, and serologic tests for antibodies against Toxoplasma gondii, Treponema pallidum, and HIV were all negative. Serial intravitreal methotrexate injections elicited an immediate reduction in EBV DNA copy levels, along with clinical improvement. Therefore, it is wondered whether the retinitis in this patient was neoplastic. A similar case was reported by Imai et al. [10] that was ultimately observed to have an intraocular natural killer (NK)/T-cell lymphoma. Although several cases of intraocular NK/T-cell lymphoma have been previously reported [11], such rare EBV-associated NK/T-cell tumors appear to be more likely to involve the orbit or ocular adnexa [12].

The abovementioned reports add to the growing number of reports identifying EBV in patients with ocular inflammation. As noted, the most convincing evidence for a direct role of EBV comes from its identification in those eyes with lymphoproliferative disorders known, in some, to be induced by EBV, including B- and NK/T-cell lymphoid tumors. However, for most patients with ocular inflammation, the presence of either EBV DNA or anti-EBV antibodies appears to be both noncausal and of limited or no clinical utility. If for some reasons ocular levels of EBV DNA or anti-EBV antibodies are assayed and observed to be elevated, consideration should be given to the possibility of an intraocular EBV-associated malignancy and, independently, HIV coinfection [13].

## 4. Conclusion

In conclusion, EBV infection might be a risk factor for PIOL.

## Data Availability

The datasets used and/or analyzed during the current study are available from the corresponding author on reasonable request.

## Abbreviations

PIOL: Primary intraocular lymphoma
EBV: Epstein-Barr virus
CNS: Central nervous system
OCT: Optical coherence tomography
SD-OCT: Spectral-domain optical coherence tomography
FAF: Fundus autofluorescence
DLBCL: Diffuse large B-cell lymphoma
PCR: Polymerase chain reaction
HSV: Herpes simplex virus
VZV: Varicella-zoster virus
CMV: Cytomegalovirus
DNA: Deoxyribonucleic acid
MRI: Magnetic resonance imaging
BCVA: Best-corrected visual acuity
CSF: Cerebrospinal fluid
HIV: Human immunodeficiency virus
NK: Natural killer
HLH: Hemophagocytic lymphohistiocytosis.
